# A Single-Cell Network-Based Drug Repositioning Strategy for Post-COVID-19 Pulmonary Fibrosis

**DOI:** 10.3390/pharmaceutics14050971

**Published:** 2022-04-30

**Authors:** Albert Li, Jhih-Yu Chen, Chia-Lang Hsu, Yen-Jen Oyang, Hsuan-Cheng Huang, Hsueh-Fen Juan

**Affiliations:** 1 Graduate Institute of Biomedical Electronics and Bioinformatics, National Taiwan University, Taipei 106, Taiwan; albert0325162@gmail.com (A.L.); a402250025@gmail.com (J.-Y.C.); yjoyang@csie.ntu.edu.tw (Y.-J.O.); 2 Department of Medical Research, National Taiwan University Hospital, Taipei 106, Taiwan; chialanghsu@ntuh.gov.tw; 3 Graduate Institute of Medical Genomics and Proteomics, National Taiwan University, Taipei 106, Taiwan; 4 Institute of Biomedical Informatics, National Yang Ming Chiao Tung University, Taipei 112, Taiwan; 5 Department of Life Science, National Taiwan University, Taipei 106, Taiwan; 6 Center for Computational and Systems Biology, National Taiwan University, Taipei 106, Taiwan

**Keywords:** single-cell RNA sequencing, COVID-19, pulmonary fibrosis, biological networks, drug repurposing

## Abstract

Post-COVID-19 pulmonary fibrosis (PCPF) is a long-term complication that appears in some COVID-19 survivors. However, there are currently limited options for treating PCPF patients. To address this problem, we investigated COVID-19 patients’ transcriptome at single-cell resolution and combined biological network analyses to repurpose the drugs treating PCPF. We revealed a novel gene signature of PCPF. The signature is functionally associated with the viral infection and lung fibrosis. Further, the signature has good performance in diagnosing and assessing pulmonary fibrosis. Next, we applied a network-based drug repurposing method to explore novel treatments for PCPF. By quantifying the proximity between the drug targets and the signature in the interactome, we identified several potential candidates and provided a drug list ranked by their proximity. Taken together, we revealed a novel gene expression signature as a theragnostic biomarker for PCPF by integrating different computational approaches. Moreover, we showed that network-based proximity could be used as a framework to repurpose drugs for PCPF.

## 1. Introduction

Since 2019, the outbreak of the COVID-19 pandemic has caused millions of infections globally. Some patients may suffer from sequelae of the viral infection [1]. Post-COVID-19 pulmonary fibrosis (PCPF) is one of the long-term complications being emphasized recently [1]. Considering the medical treatments for this disease are limited, it is crucial to leverage pharmacogenomic data to repurpose drugs treating this disease. In this study, we combine single-cell analysis, machine learning, and network biology to identify a novel transcriptomic signature. We show that this signature is promising in assessing the disease and surveying drugs that can potentially treat pulmonary fibrosis.

Previously, network-based methods have successfully repurposed drugs treating several diseases [2,3,4,5]. Based on the property of biological networks, drugs with smaller proximity tend to be more effective than those with larger proximity [3]. However, since the choice of disease-related genes will largely impact results and inferences [6], whether the network-based approach can be applied to PCPF needs further verification.

Single-cell RNA-sequencing analysis (scRNA-seq) has been used to investigate the host response in severe COVID-19 cases [7]. Melms et al. discovered that two cell types, pathological and intermediate-pathological fibroblasts, are associated with the pathogenesis of pulmonary fibrosis; these cells strongly express markers of pathological fibroblasts (*CTHRC1*) and pathological extracellular matrix (*COL1A1* and *COL3A1*) [7]. They also revealed a clear relationship between fibrosis score and mortality, highlighting the importance of pulmonary fibrosis in patients’ survival. Although the roles of pathological fibroblasts have been elucidated, whether these cells are applicable in clinical diagnosis, severity assessment, and treatment still needs further investigation.

Here, we aim to reveal a novel signature of PCPF by interrogating scRNA-seq data. We showed that the signature could be used to diagnose and assess pulmonary fibrosis. Further, this signature can also be used to repurpose and prioritize potentially effective drugs treating PCPF.

## 2. Materials and Methods

### 2.1. Construction and Evaluation of the PCPF Signature

The preprocessed single-cell gene expression profile underwent linearly dimensional reduction by principal component analysis (PCA). We used the Louvain algorithm to cluster the cells on the K-nearest neighbors (KNN) graph, which was constructed on the principal component (PC) space. We referred to the cell (sub)type information provided by Melms et al. [7]. We annotated each cell cluster based on the majority of the cell subtype in each cluster. Next, we made a case-control comparison to calculate the proportion difference in different cell clusters. To identify the characters of the cluster with the greatest proportional changes, we conducted differential gene expression analysis to compare the gene expression profiles of the cases and controls. We selected the top 200 up-regulated differentially expressed genes (DEGs) as the PCPF signature. We defined the signature score as the mean of the signature gene expression. We implemented the single-cell analysis with Scanpy [8].

We used DAVID (Available online: https://david.ncifcrf.gov/; (accessed on July 2021)) [9] to infer the signature-related biological functions. We selected the Benjamini−Hochberg procedure for the adjustment of multiple hypothesis testing.

### 2.2. Support Vector Machine (SVM)

Samples from GSE32537 underwent a random selection where 80% of samples were used for model training and the remainder for testing. A non-linear decision boundary, radial kernel function, was used to maximize the margin *M* that delineates two different classes (i.e., cases and controls). Ten-fold cross-validation was used to select optimal tuning parameters *C* and γ, where *C* determines the tolerance of violation to the margin and γ defines how far the support vectors should be taken. We compared the SVM values between cases and controls in the testing dataset (Wilcoxon rank-sum test). The procedure was implemented with the R package e1071.

### 2.3. Principal Component Regression

Observations from GSE32537 underwent random sampling where 2/3 of samples were used for model training, and the remaining samples were used for testing. Expression levels of genes within the signature were dimensionally reduced to PCs. We used PCs as features to predict DLCO and FVC. Suppose there are *m* observations, **y** represents the response vector in $R^{m}$, and *n* is the total number of PCs. We composed a design matrix $P_{m\;\times \;\left(k+1\right)}$ with a constant column and the first *k* PCs, and fitted a linear regression model as:

(1) y=Pβ+ϵ

With the lowest loss (mean square error, MSE), where $\beta \in R^{k+1}$ is the coefficient vector, $\epsilon \in R^{m}$ is the error vector, and k∈[1,n]. Ten-fold cross-validation was used to assess the models for different *k*. Since the cut-offs of abnormal DLCO and FVC (% predicted) are typically set at 75% and 80% [10], respectively, we filtered out samples beyond those thresholds. The testing dataset was used to predict clinical traits (DLCO and FVC). Correlation analysis (Pearson’s r) was conducted to assess the association between predicted and observed values. We implemented the procedure with the R package *pls* [11].

### 2.4. Calculation of Network-Based Proximity

Proximity is the shortest path length between two sets of nodes (drug targets and disease-related proteins) in the interactome. Suppose that *T* is the set of protein target(s) of a drug, *D* is the set of proteins relating to the disease, and l(t,d) is the shortest path length between node *t* and *d*. Therefore, the shortest proximity (ds) is defined as follows:

(2) $$\mathrm{ds}=\frac{1}{\left|\left|T\right|\right|}\underset{t\;\in T}{\sum }\frac{1}{\left|\left|D\right|\right|}\underset{d\;\in \;D}{\sum }l\;\left(t,d\right)\;\forall \;t\in T,\;d\in D\;$$

To reduce the degree effect in proximity, we calculated the relative proximity Zds by stratifying the nodes according to their degrees. Specifically, nodes in the interactome were firstly arranged according to node degree and assigned to bins sequentially, where each bin can at most contain 100 nodes. Here, nodes in each bin will have similar, if not identical, degrees. Second, we randomly selected nodes from the same bin as nodes in the set *T* and *D*, then computed their shortest proximity. The procedure was iterated 100 times to obtain the mean (μds) and standard deviation (σds) of ds. The relative proximity (Zds) is defined as:

(3) $$\mathrm{Zds}=\frac{\mathrm{ds}-\mu \mathrm{ds}}{\sigma \mathrm{ds}}$$

## 3. Results

### 3.1. An Overview of the Analytical Pipeline

**Figure 1:**
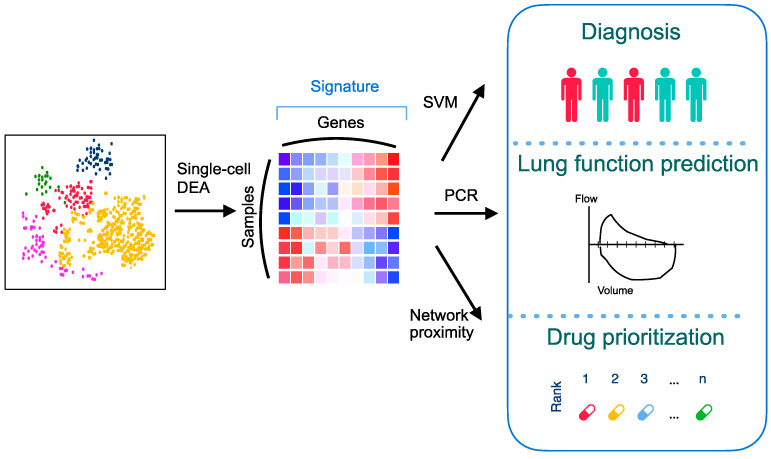
An overall analytical pipeline of this study. Schematic representation of the scRNA-seq analysis, signature construction, and application of the signature by integrating various computational methods. DEA: differential expression analysis; PCR: principal component regression; SVM: support vector machine.

The aims of this study are to discover a novel PCPF signature and leverage the network-based drug repurposing method to explore medications treating PCPF. The analytical pipeline is shown in Figure 1. We first identify the cell (sub)types and annotate cell clusters. We next construct the PCPF signature and evaluate its roles in diagnosing and assessing pulmonary fibrosis. Finally, we use a network-based method to explore effective treatment for PCPF.

### 3.2. Identifying PCPF-Related Cell Clusters at the Single-Cell Level

**Figure 2:**
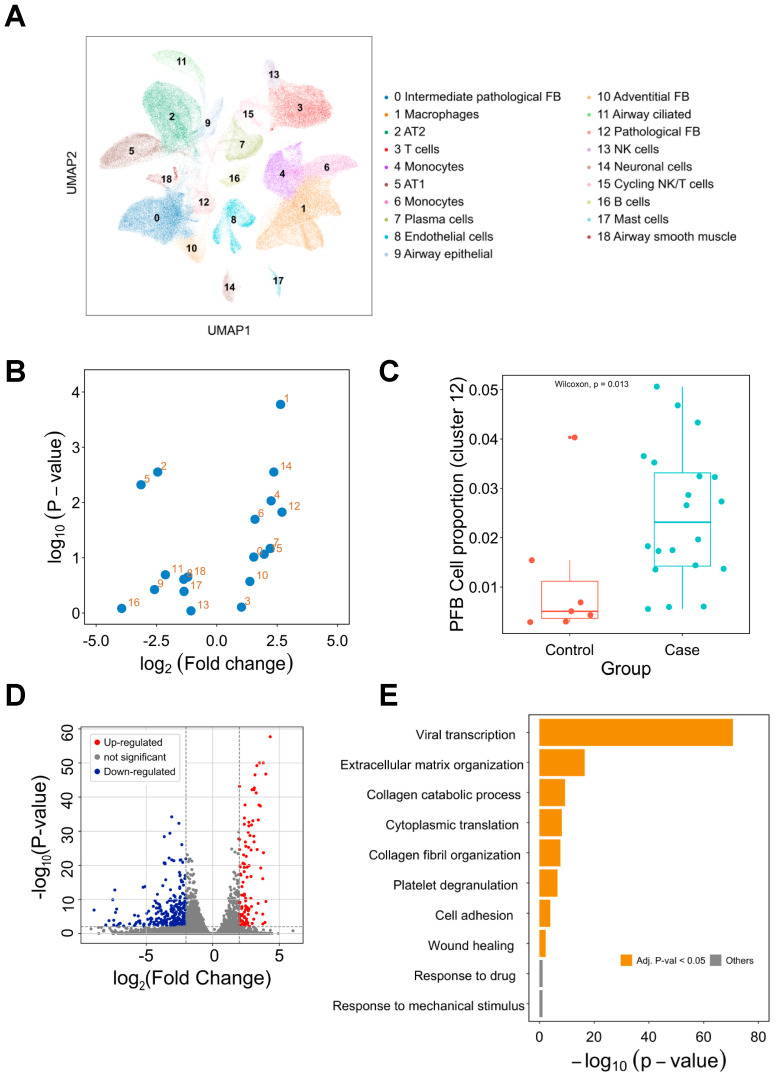
Single-cell transcriptome analysis of the lung tissues in COVID-19 cases. (**A**) Single-cell analysis of 116,314 cells from lung tissues. Nineteen cell clusters were identified and annotated based on the cell (sub)types provided by the literature [7]. (**B**) Visualization of the proportional difference of cells between COVID-19 patients and healthy controls. (**C**) Comparison of cluster 12 (PFBs) proportion between COVID-19 patients and healthy controls. (**D**) Differentially expressed gene analysis of cluster 12. Up-regulated and down-regulated genes are highlighted in red and blue, respectively. (**E**) Functional enrichment analysis of the differentially expressed genes. Enriched biological processes are shown in a bar plot. pFB: pathological fibroblast. PCPF: post-COVID-19 pulmonary fibrosis.

To explore cell clusters contributing to PCPF, we first investigated lung tissues on the dimensionally-reduced 2D plane (Figure 2A). To discover which cell cluster is mainly associated with PCPF, we conducted a case-control comparison on each cell cluster to compare their proportional differences (Figure 2B). We then noticed that cluster 12, pathological fibroblasts (PFBs), has the most considerable difference (Figure 2C). Therefore, we posited that PFBs play crucial roles in PCPF pathogenesis and further explored their clinical impact.

### 3.3. Comparison of Pathological Fibroblasts (PFBs) to Other Cell Types

To deduce the roles of PFBs in PCPF, we compared the gene expression profile between PFBs and other cells (Figure 2D and Supplementary Figure S1). To infer the biological functions in which DEGs are involved, we performed a functional enrichment analysis to identify the enriched biological processes (BP) in PFBs (Figure 2E). We found that viral transcription is the most enriched term, followed by fibrosis formation (e.g., extracellular matrix organization and collagen fibril organization). The DEGs derived from PFBs show meaningful and related biological functions, suggesting that PFBs may contribute to PCPF pathogenesis. Therefore, we constructed a transcriptome signature (Supplementary Table S1) to represent the distinct expression profile of these PFBs and further explored the roles of the signature on pulmonary fibrosis patients’ outcomes.

### 3.4. Difference in PFB Signature between the Patients and Healthy Controls

**Figure 3:**
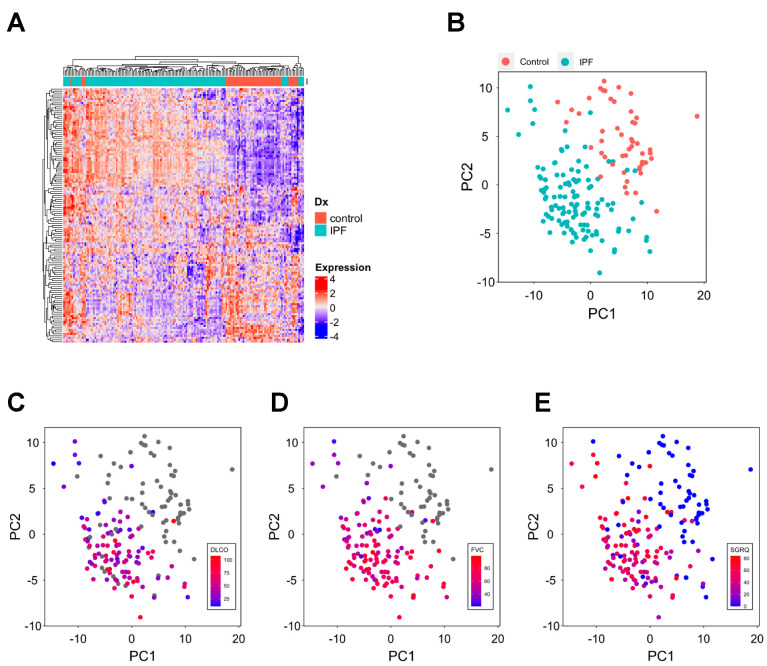
Discovery of distinct expression of the signature in pulmonary fibrosis patients. (**A**) Hierarchical clustering of samples based on the signature expression. Heatmap values are the scaled gene expression. (**B**) Visualization of patients and controls in the two main principal components. (**C**–**E**) Visualization of DLCO (**C**), FVC (**D**), and SGRQ (**E**) in the two main principal components. DLCO: diffusing capacity for carbon monoxide; FVC: forced vital capacity; SGRQ: St. George’s Respiratory Questionnaire.

To further discover the signature derived from the scRNA-seq of COVID-19 samples, we externally validated the PFB signature in another cohort, comprising 119 idiopathic pulmonary fibrosis (IPF) patients and 50 healthy controls [12]. IPF patients and healthy people have a distinct signature pattern (Figure 3A,B). Next, we examined whether patients’ symptoms (SGRQ) and lung function (FVC and DLCO) could be clearly visualized within the two main PCs as well. DLCO and FVC show an increasing trend from the top left to the bottom in the first two principal component dimensions (Figure 3C,D), suggesting that patients with different IPF severity are dissimilar in terms of their signature. Although not as clear as that in lung function, the SGRQ trend is also similar, where more severe patients appeared in the top left, and less impaired patients appeared in the bottom right (Figure 3E).

### 3.5. The Signature Can Be Used in the Diagnosis and Severity Assessment of Pulmonary Fibrosis

**Figure 4:**
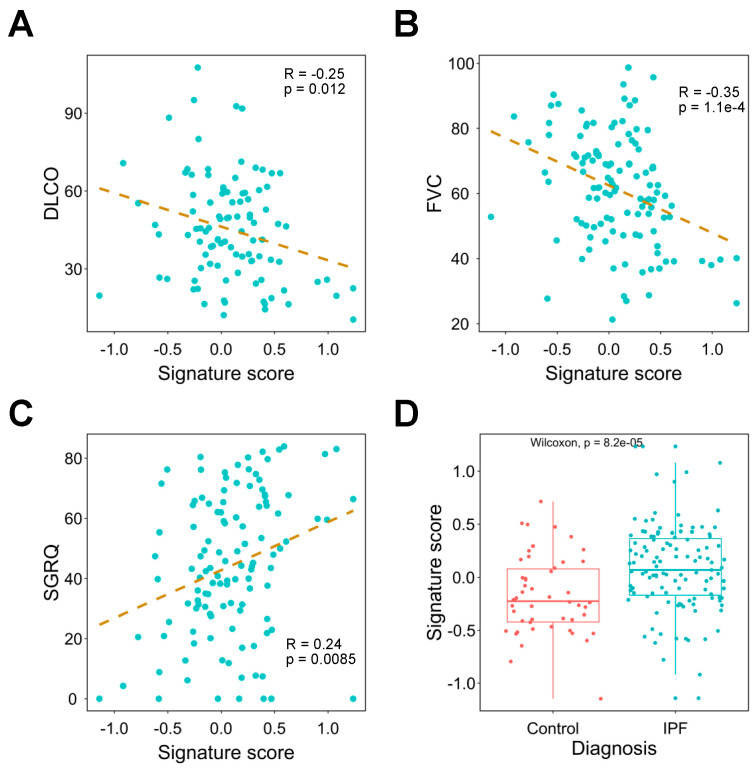
Investigating the association between signature expression and lung functions. (**A**–**C**) Correlation analysis of signature score and DLCO (**A**), FVC (**B**), and SGRQ (**C**). The dashed line represents the linear regression line. (**D**) Comparison of signature expression between IPF patients and healthy controls. DLCO: diffusing capacity for carbon monoxide; FVC: forced vital capacity; IPF: idiopathic pulmonary fibrosis; SGRQ: St. George’s Respiratory Questionnaire.

Current genetic tools for the diagnosis and assessment of pulmonary fibrosis are limited. Therefore, we explored whether the signature can be applied to these clinical challenges. We first revealed that FVC, DLCO, and SGRQ are significantly correlated with the signature score (Figure 4A–C). Moreover, as a potential confounder of clinical traits, age has a very weak correlation with SGRQ, FVC, and DLCO (Supplementary Figure S2). Next, we compared signature scores between IPF patients and healthy people and found IPF patients have significantly higher scores compared to the controls (Figure 4D).

**Figure 5:**
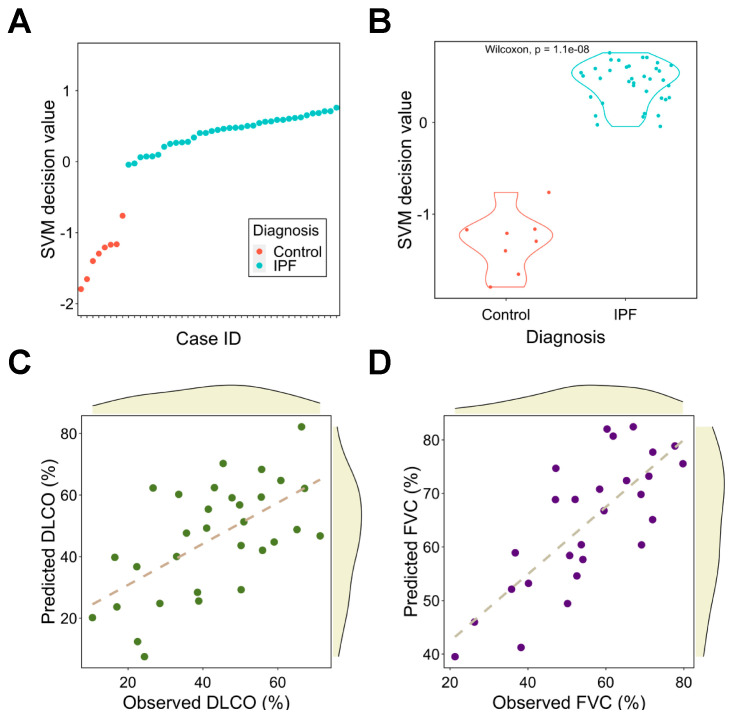
Signature as a diagnosis and assessment tool for pulmonary fibrosis using machine learning models. (**A**) The SVM scores for IPF patients and healthy controls. (**B**) Comparison of SVM decision value between IPF patients and healthy controls. (**C**,**D**) Correlation analysis between observed and predicted DLCO (**C**) and FVC (**D**). The dashed line represents the linear regression line. DLCO: diffusing capacity for carbon monoxide; FVC: forced vital capacity; IPF: idiopathic pulmonary fibrosis; SVM: support vector machine.

Considering the correlation between gene signature and traits, we next used the signature to train machine learning models to predict clinical outcomes of pulmonary fibrosis patients. We found that an SVM could perfectly differentiate pulmonary fibrosis patients from healthy controls (Figure 5A,B) without adding extra clinical features. We next explored whether the signature could predict patients’ lung function test results (% of predicted DLCO and FVC). PC regression was used to fit the training data. The correlation coefficients between the predicted and observed DLCO and FVC are 0.61 (*p* = 2.91 × 10^−4^) and 0.77 (*p* = 2.52 × 10^−6^ respectively (Figure 5C,D).

Altogether, the signature has high confidence in classifying pulmonary fibrosis patients and predicting lung function test results; this implies its potential applicability in clinical diagnosis and severity assessment.

### 3.6. The Network-Based Proximity between Anti-Pulmonary Fibrosis Drugs and the Signature

Considering the roles of the signature in the diagnosis and assessment of pulmonary fibrosis, we defined the top-20 genes in the signature as the disease-related genes. Since the network proximity has been used to evaluate drugs for various diseases [3,4], we postulated that this method could also prioritize and repurpose the anti-PCPF drugs. In this case, anti-pulmonary fibrosis drugs should have closer proximity than the drugs with unknown anti-pulmonary fibrosis effects.

**Figure 6:**
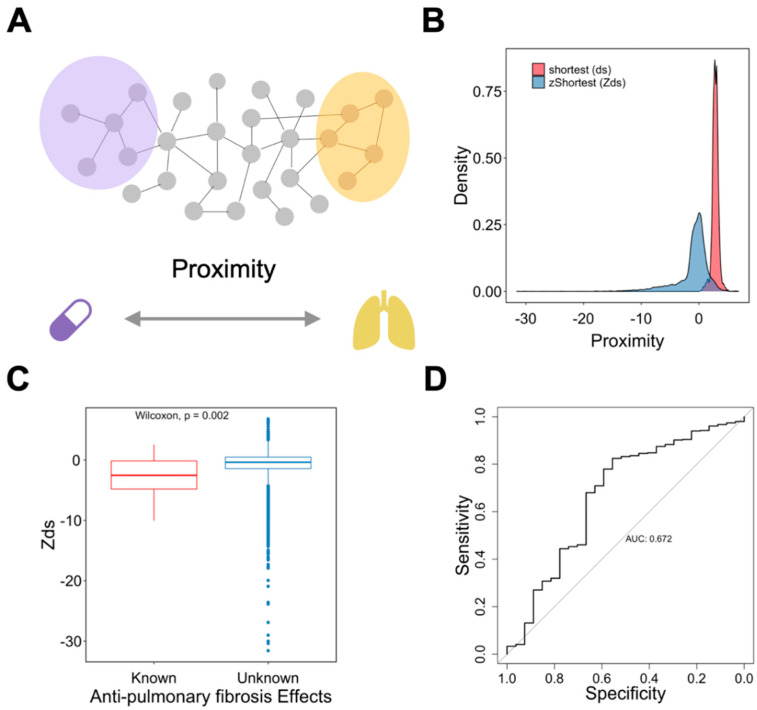
Characterizing the roles of proximity on drug repurposing for anti-pulmonary fibrosis drugs. (**A**) Schematic representation of the method. (**B**) Distribution of different proximity measures. (**C**) Comparison of proximity, Zds, between drugs with known and unknown anti-pulmonary fibrosis effects. (**D**) Analysis of the predictive performance of Zds on anti-pulmonary fibrosis effects using the ROC curve.

We calculated the shortest proximity (ds) between drug targets and PCPF-related proteins on the interactome (Figure 6A). Since our hypothesis is that shorter proximity is associated with therapeutic effects, it is necessary to examine other factors that simultaneously affect proximity. In particular, node degree has been known to be anti-correlated with proximity [3], defined here as degree effect. Degree effect can lead to a biased interpretation of proximity in drug repurposing analyses. For instance, the cytotoxic agents typically have lower proximity than other drug categories because anti-cancer drugs’ targets tend to have higher node degrees [2]. In this study, we also observed this phenomenon (Supplementary Figure S3A,B). We then calculated the relative proximity (Zds) by randomly selecting the degree-stratifying nodes on the interactome (Figure 6B). It is clear that the degree effect is less prominent in Zds (Supplementary Figure S3C,D). Next, to prove that the known-effect (anti-pulmonary fibrosis) drugs have smaller proximity than the unknown-effect drugs, we compared Zds between these two categories. We found that the known-effect drugs have significantly lower proximity (Figure 6C), with predictive performance AUC equal to 0.672 (Figure 6D). To further validate the results, we used another set of anti-fibrosis drugs (not restricted to pulmonary fibrosis) [13] and found identical trends (Supplementary Figure S4A,B). Based on the above results, Zds can be used as a predictor to assess anti-pulmonary fibrosis effects. Therefore, we summarized the drugs with high repurposing potential in Table 1. The full drug list and their proximity information can be found in Supplementary Table S2.

## 4. Discussion

This study integrates various computational approaches to reveal a crucial theragnostic signature in PCPF. We show that the signature is associated with viral infections, pulmonary fibrosis, and clinical outcomes. Moreover, we demonstrate that the machine learning models trained with the signature show decent performance in diagnosing pulmonary fibrosis and predicting patients’ lung function. Lastly, we prove that drugs with known anti-pulmonary fibrosis effects have closer proximity than those with unknown effects, suggesting that a network-based framework can also be applied to prioritize and repurpose drugs in PCPF.

Considering the design of this study was for PCPF, we notice that the viral infection-related GO term is the most enriched (Figure 2E). This phenomenon also appears in the network-based analysis, where drugs with strong anti-COVID-19 effects have significantly closer (smaller) proximity than drugs with weak or no-effect (Supplementary Figure S3D). This observation suggests that the signature may be associated with two events: COVID-19 viral infection and pulmonary fibrosis. Although pulmonary fibroblasts are less well known as target cells of the virus, recent studies revealed that alveolar fibroblasts could also be infected by the virus due to their expression of ACE2 receptors [28]. Aloufi et al. found that IPF fibroblasts have an even higher expression of ACE2 receptor, highlighting the roles of pathological fibroblasts in COVID-19 infection [29].

We also observe some medical procedure-related terms (e.g., response to mechanical stimulus). Although these terms are not significantly enriched (Figure 2E), they still imply that patients may undergo specific medication therapies or receive mechanical ventilation during hospital treatment.

One of the advantages of performing scRNA-seq on clinical samples is the high-resolution mapping of each cell. However, a deeper inspection may imply a smaller patient sample size because the number of patients enrolled can rarely be as large as that in bulk RNA analysis. There are 26 cases in the scRNA-seq dataset; it is reasonable to challenge any inference made from only 26 persons. Therefore, externally validating the results derived from scRNA-seq in a broader population can generate more confidence in the results. Nonetheless, it is undeniable that some facts exist such that the results from scRNA-seq may not be fully in concordance with bulk RNA analysis. Zero inflation, for instance, can lead to the underestimation of the low-expressed genes [30]. Another challenge is that the result in one patient cohort may not be reproducible in another simply due to numerous uncontrollable factors between the two cohorts. However, in our study, the signature derived from scRNA-seq also play a vital role in another bulk-sample patient cohort, suggesting that the signature is reproducible and can be externally validated.

There are limitations to this study. First, we applied the signature derived from PCPF to IPF patients. It is undeniable that the etiologies of PCPF and IPF are less likely to be identical. The causes of PCPF may include the viral infection and the host immune response; on the other hand, the causes of IPF remain unclear, even though there are several studies revealed the genetic predispositions or causal variants of IPF using genome-wide association studies with fine-mapping [31] or polygenic risk score [32]. However, regardless of the causes, PCPF and IPF are fibrogenesis and fibrosis in the lung tissue. Considering the limited clinical information on PCPF, we used IPF as a surrogate to investigate the potential impacts and clinical insights of this PCPF signature, in particular the application in drug repurposing. We understand that population structure and other bassline demographic characteristics could influence the performance of the gene signature score, and thus the signature score should be carefully interpreted when applying to other ethnic groups, such as Asians. Another limitation is the lack of lung function test results in the single-cell cohort. This makes it harder to compare the baseline characteristics of the IPF and PCPF patients.

The rationale for the network-based drug repurposing approach is that a drug may still be effective when its target proteins are ‘close’ to the disease-related protein(s) in the interactome [3,33,34]. If this argument is true, drugs with known effects on disease should have closer proximity compared to the unknown-effect drugs. Accordingly, this requires identifying a significant difference in proximity between known-effect and unknown-effect drugs. However, in some diseases, medical treatment options are very limited, such as IPF [35,36]. There are, in fact, only two FDA-approved drugs, nintedanib, and pirfenidone, that seem to be associated with a slower progression of IPF [36]. Therefore, if we simply assign drugs to either known or unknown effects based on current clinical knowledge, hypothesis testing between the two drug categories (known vs. unknown effect) can hardly be conducted due to highly unbalanced sample sizes. To address this problem, we searched the published literature which conducted drug repurposing for pulmonary fibrosis [37] and pan-fibrosis [13] and used the repurposed drugs as the known-effect drugs.

Previous studies have applied the network-based drug repurposing framework to various diseases [3,38]. Nonetheless, due to the complexity of disease mechanisms, validating this method is necessary when dealing with different conditions. For instance, previously, we found that, in lung adenocarcinoma, the closest proximity on the weighted interactome shows the best performance in identifying promising drugs [2]. In this study, however, we noticed that z-transformed shortest proximity, Zds, has better performance. This observation implies that the performance of proximity metrics may be context-dependent.

Although proximity may be associated with drug effectiveness, we urge caution when interpreting the ranked drug list, as proximity is not the only factor contributing to drug effectiveness. For instance, we found that nintedanib, one of the two currently approved drugs for IPF, has small proximity (Zds = −3.22; rank = 798/5643). However, the other approved anti-IPF agent, pirfenidone, has large proximity (Zds = 1.45; rank = 5115/5643). Therefore, this observation suggests that drugs with distant proximity could still be effective, as proximity may be only one of the many factors affecting drug effectiveness. Other crucial factors, such as binding affinity, also matter.

Within the top-ranked repurposed drugs (top 3% of the drugs in Supplementary Table S2), we found some drugs belonging to antibiotic or antiviral agent categories, which may be related to pneumonia treatment [39], acute exacerbation of pulmonary fibrosis [40], or other morbidities, such as pneumonitis, opportunistic infection, or tissue inflammation [41]. They may not truly show strong anti-fibrosis effects. On the other hand, we noticed that many top-ranked candidates on this list show promising anti-pulmonary fibrosis effects. Artenimol (Zds = −14.18; rank = 28/5643) (also known as dihydroartemisinin), for instance, can reduce lung fibrosis by suppressing the Notch signaling pathway [42] and pro-fibrotic pathways [43]. Another example is dinoprostone (also known as prostaglandin E2). It was reported that inhaling liposomal prostaglandin E2 can treat pulmonary fibrosis by restricting inflammation and fibrotic injury in the lungs [21].

Another interesting drug category is statins, a well-known class of lipid-lowering agents. A retrospective study surveying 323 IPF patients found that statin-users have a slower annual decline in DLCO and FVC than non-users [20]. We then searched our drug list for the types of the statin used in this study [20] and found that all of them have very small Zds: atorvastatin (Zds = −10.5), fluvastatin (Zds = −10.03), rosuvastatin (Zds = −8.37), pravastatin (Zds = −6.73), and simvastatin (Zds = −4.43).

## 5. Conclusions

We reveal a novel theragnostic signature for PCPF and provide a prioritized drug list based on network-based proximity, Zds. Our study shows the applicability of integrating various computational methods when analyzing biomedical data and, importantly, provides useful information for diagnosing, assessing, and treating PCPF.

## Figures and Tables

**Table 1:** Selected top-ranked drugs with highly anti-pulmonary fibrosis potential.

Name	Z-Shortest Proximity (Zds)	Shortest Proximity (ds)	Structure	Reference
Benzoic Acid	−17.91	0.726	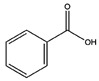	[14,15]
Artenimol	−14.18	2.019	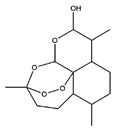	[16]
Quercetin	−12.48	2.060	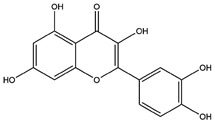	[17]
Tauroursodeoxycholic acid	−11.73	0.783	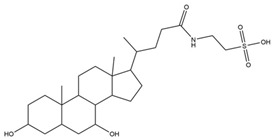	[18]
Atorvastatin	−10.51	2.323	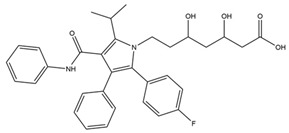	[19,20]
Dinoprostone	−10.45	2.376	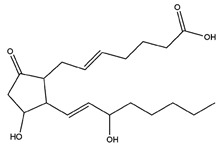	[21]
Emodin	−10.25	1.238	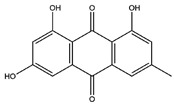	[22]
Valproic Acid	−10.11	2.373	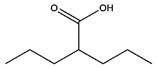	[23]
Fluvastatin	−10.03	2.379	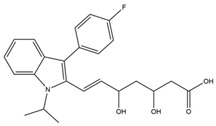	[20]
Cerulenin	−10.03	0.688	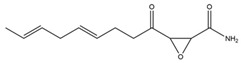	[24]
Naringenin	−9.40	2.204	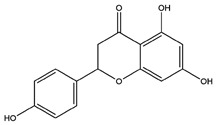	[25]
Fisetin	−9.18	1.325	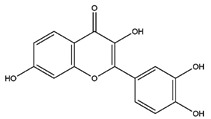	[26]
Vitamin D	−9.18	1.690	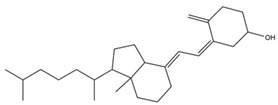	[27]

## Data Availability

The COVID-19 scRNA-seq dataset was derived from Melms et al. [7]. It contains autopsy lung tissues from 26 patients, containing 116,314 cells. The pulmonary fibrosis cohort was downloaded from GEO (GSE32537) [12], which provided the gene expression profiles and clinical traits of 119 idiopathic pulmonary fibrosis (IPF) patients and 50 healthy controls. The clinical traits include St. George’s Respiratory Questionnaire (SGRQ) and lung function test results (diffusing capacity for carbon monoxide (DLCO) and forced vital capacity (FVC)). For the proximity calculations, we adapted human protein–protein interaction data from Guney et al. [3], which comprises 140,637 interactions among 13,101 proteins. We retrieved and adapted drug-related information, including drug targets and their anti-SARS-CoV-2 effects, from the Drugbank database [44] and Gysi et al. [4], respectively.

## Supplementary Materials

The following are available online at https://www.mdpi.com/article/10.3390/pharmaceutics14050971/s1, Figure S1: The expression of DEGs in PCPF patients and the controls, Figure S2: The correlation analysis between age and other clinical features, Figure S3: The degree effect on proximity, Figure S4: Characterizing the roles of proximity on the repurposing of anti-fibrosis drugs, Table S1: The transcriptome signature of pathological fibroblasts in PCPF, Table S2: The full list with 5644 drugs and their proximity (Zds).
