# Supplementary material for: A Single-Cell Network-Based Drug Repositioning Strategy for Post-COVID-19 Pulmonary Fibrosis
Source: Pharmaceutics. 2022 Apr 30;14(5):971. doi: 10.3390/pharmaceutics14050971 (PMC9147547; doi:10.3390/pharmaceutics14050971)
Supplement: Supplementary file 1 [file pharmaceutics-14-00971-s001.zip › Supplementary information.pdf]

Supplementary information

A single-cell network-based drug repositioning strategy for post-COVID-19 pulmonary fibrosis

Albert Li, Jhih-Yu Chen, Chia-Lang Hsu, Yen-Jen Oyang, Hsuan-Cheng Huang, Hsueh-Fen Juan

The supplementary data includes:

1. Supplementary Figures

Supplementary Figure S1. The expression of DEGs in PCPF patients and the controls

Supplementary Figure S2. The correlation analysis between age and other clinical features

Supplementary Figure S3. The degree effect on proximity.

Supplementary Figure S4. Characterizing the roles of proximity on the repurposing of anti-fibrosis drugs.

2. Supplementary Tables

Supplementary Table S1. The transcriptome signature of pathological fibroblasts in PCPF.

Supplementary Table S2. The full list with 5644 drugs and their proximity (Zds). (A separate excel file).

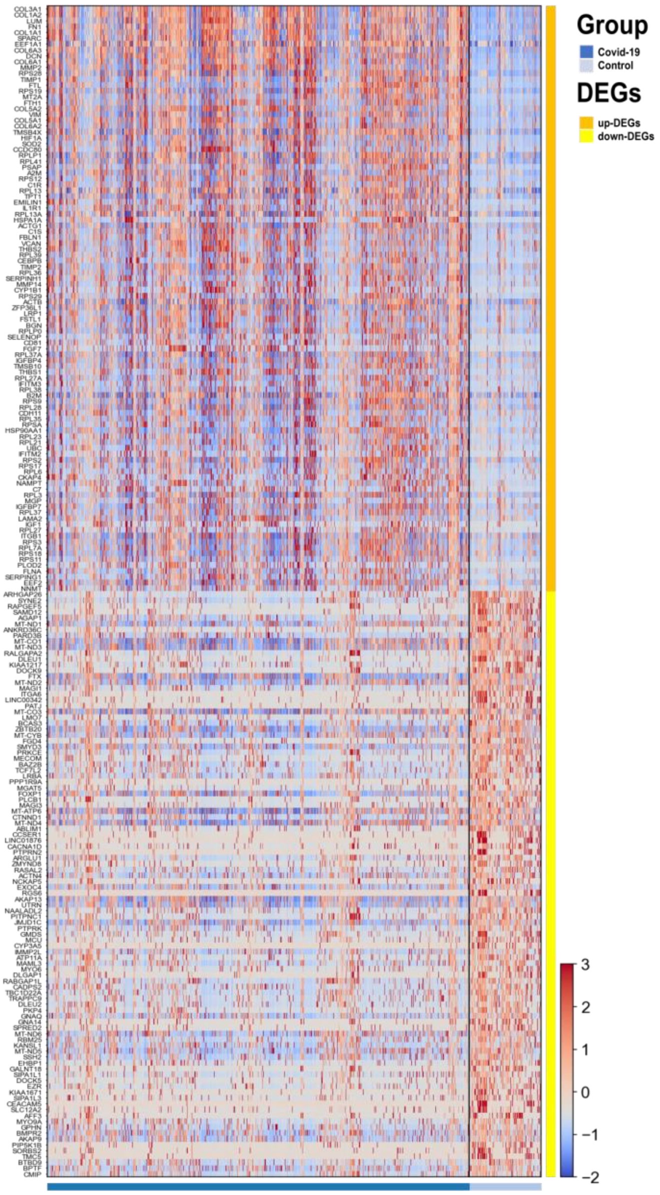

Supplementary Figure S1. Visualization of the expression of the up- and down-regulated differentially expressed genes. The values in the heatmap represent scaled gene expression.

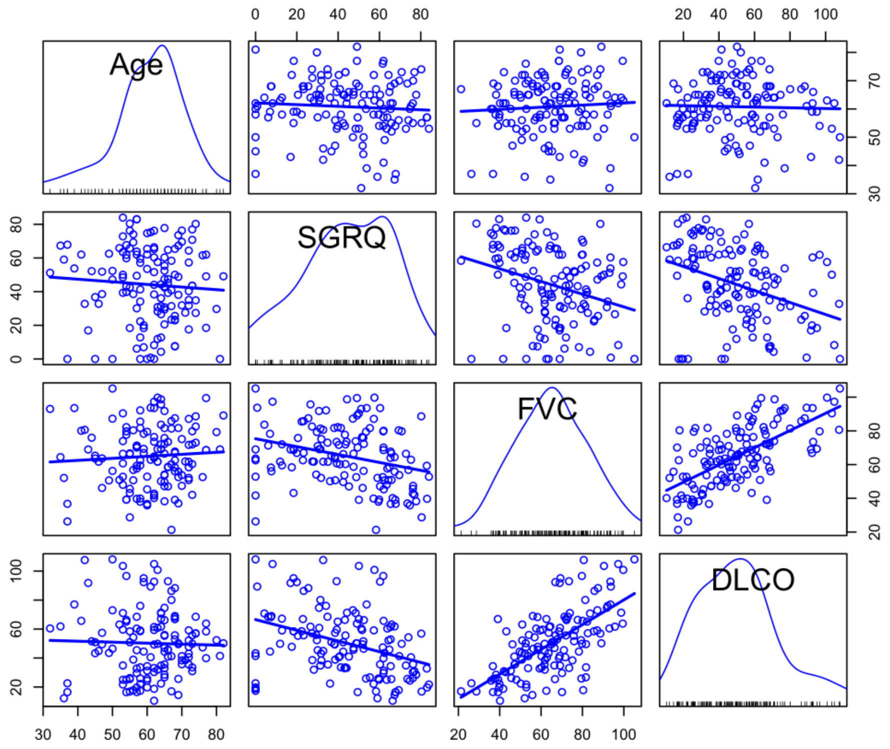

Supplementary Figure S2. The correlation analysis between age and other clinical features: SGRQ, FVC, and DLCO.

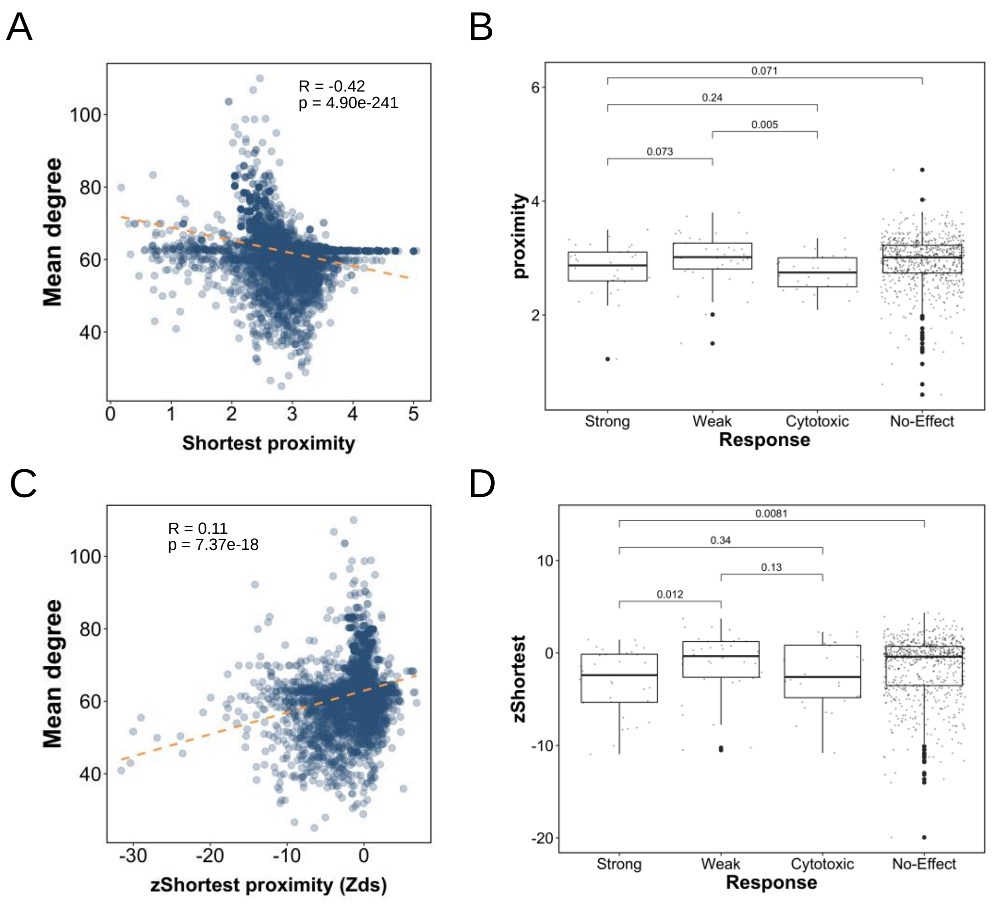

Supplementary Figure S3. The degree effect on proximity. (**A**) Correlation analysis of node degree and shortest proximity. (**B**) Comparison of shortest proximity in different drug categories. (**C**) Correlation analysis of node degree and z-transformed shortest proximity (Zds). (**D**) Comparison of Zds in different drug categories.

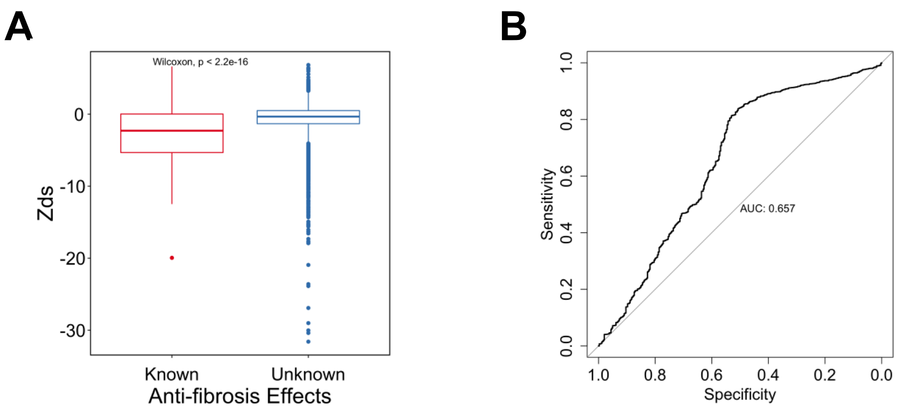

Supplementary Figure S4. Characterizing the roles of proximity on the repurposing of anti-fibrosis drugs. (**A**) The comparison of proximity, Zds, between drugs with known and unknown anti-fibrosis effects. (**B**) Analysis of the predictive performance of Zds on anti- fibrosis effects using a ROC curve.

Supplementary Table S1. The transcriptome signature of pathological fibroblasts in PCPF.

COL3A1	COL6A2	VCAN	IGFBP4	CKAP4	HSPA1B	RARRES2	RPS24	COL14A1	AEBP1
COL1A2	TMSB4X	THBS2	TMSB10	NAMPT	LAPTM4A	RPL18	RPS27A	FAP	NUPR1
LUM	HIF1A	RPL39	THBS1	C7	RPS27	RPL29	RPS14	RPS25	LAMP2
FN1	SOD2	CEBPB	RPL27A	RPL3	HSPB1	RPS6	GNAS	SERPINF1	MXRA5
COL1A1	CCDC80	TIMP2	IFITM3	MGP	PTGDS	CFH	COL4A1	TUBA1B	RPL30
SPARC	RPLP1	RPL36	RPL38	IGFBP7	RPL35A	ANXA5	ELL2	MYL6	S100A10
EEF1A1	RPL41	SERPINH1	B2M	RPL37	RPL9	PCOLCE	PPIB	RPL34	CXCL12
COL6A3	PSAP	MMP14	RPS9	LAMA2	HSP90B1	ADAM12	LOX	PIEZO2	RPS7
DCN	A2M	CYP1B1	RPL28	IGF1	SPON1	MAN1A1	RPS23	EFEMP1	CRTAP
COL6A1	RPS12	RPS29	CDH11	RPL27	TNC	P4HB	MRC2	LTBP2	RPL12
MMP2	C1R	ACTB	RPL35	ITGB1	RPL17	NR2F1	TXNDC5	GXYLT2	ADAMTS2
RPS28	RPL13	ZFP36L1	RPSA	RPS3	RPLP2	RPL15	RPS8	RPL32	CEBPD
TIMP1	TPT1	LRP1	HSP90AA1	RPL7A	FBN1	CTHRC1	CALU	METTL7A	CALR
FTL	EMILIN1	FSTL1	RPL23	RPS18	MOXD1	ATP5F1E	CCN1	PTMS	PTK7
RPS19	IL1R1	BGN	RPL21	RPS11	TGM2	C11orf96	CST3	FNDC3B	ABCA1
MT2A	RPL13A	RPLP0	UBC	PLOD2	PRRX1	IL6ST	RPL8	OAZ1	SCARB2
FTH1	HSPA1A	SELENOP	IFITM2	FLNA	FKBP10	PDGFRA	VGLL3	SERPINE1	PLXDC2
COL5A2	ACTG1	CD81	RPS2	SERPING1	COL15A1	HSP90AB1	CDKN1A	ROR2	ZMAT3
VIM	C1S	FGF7	RPS17	EEF2	LGALS1	CRISPLD2	NBL1	HMCN1	SQSTM1
COL5A1	FBLN1	RPL37A	RPL6	NNMT	NFIX	RPS13	FBLN2	RPL18A	GAPDH
